# Smartphone obsession linked behavioural changes among Indian adolescents

**DOI:** 10.6026/973206300191025

**Published:** 2023-10-31

**Authors:** R Ambiha, J Sathyapriya, N Akila, R Vanitha, K Muniyammal

**Affiliations:** 1 Nootan College of Nursing, Sankalchand Patel University, Visnagar, Gujarat - 384315, India; 2 Dhanalakshmi Srinivasan College of Nursing, Dhanalakshmi Srinivasan University, Perambalur, Tamil Nadu - 621212, India

**Keywords:** Smart phone obsession, behavioral changes, mobile phone addiction, adolescent's health

## Abstract

The best technological gifts of the 21st century are mobile phones, which are especially well-liked to adolescents. Infinite resources with numerous uses and applications are available on modern mobile devices. Adolescents have behavioral changes as a result of its excessive use. Therefore, it is of interest to explore the connection between smartphone obsession and potential behavioral changes. A School based, co relational study was conducted among 100 adolescents at selected schools at Tamil Nadu. Samples were selected by simple random sampling technique. Data were collected to assess the mobile addiction by using Smartphone Addiction Scale - Short Version (SVSAC) and Pediatric symptom checklist - 17was used to evaluate the behavioral changes. Collected data were analyzed by using descriptive and inferential statistics. The Study shows that 52 adolescents had a high smartphone Obsession, in that 37 had negative behavioral changes due to overuse of the smart phone and also had slight positive correlation between smartphone obsession and behavioral changes. A significant relationship was seen between smart phone obsession with behavioral changes (p<0.001) among adolescents. The study concluded that smart phone obsession and behavioral changes having strong connection in adolescents was significantly associated with their behaviors.

## Background:

Since long ago, communication has had a significant impact on our civilization. Over time, its tools and equipment have improved, enabling us to communicate with others more quickly and easily. A smartphone has recently taken the top spot among communication devices in people's daily lives. Regardless of age, gender, or area, mobile phone improvements from simple, basic phones to featured phones and smartphones led to the spread of technology among various categories of people [1]. Smartphone use has increased significantly among today's children and young people during the past ten years, concurrently with an uptick in mental illness in this population. At the same time, media attention is focused on the possibility of "smartphone addiction" or inappropriate smartphone use [2]. The rise in human-machine interactions is largely due to smartphones, which has several benefits. But as smartphone use becomes more widespread, it has also resulted in addiction and misuse [3]. In the highly computerized modern environment that defines the 21st century, smartphones have integrated themselves into the daily lives of adolescents. In addition to their many benefits, smartphones could lead to excessive usage and addictive behaviours [4]. Smartphone addiction has been linked to physical health issues, which can result in sleep abnormalities, musculoskeletal issues, and neurological issues. Additionally, smartphone addiction was significantly correlated with poor academic performance, procrastination, impulsivity, self-esteem, decreased social contact, solitude, and suicide [5]. Since many years ago, studies on smartphone use and its effects on all teenagers have been conducted. It is by no means a recent problem. However, teen psychological and physical health is declining, and cell phone addiction is on the rise. Some researcher examined psychological behavior and social relationships with mobile phone addiction. Some researchers looked at adolescents' physical health or academic performance with smartphone addiction [6]. The scores from the Smartphone Addiction Proneness Scale indicated 1261 (69.1%) as the usual user group and 563 (30.9%) as a risk group for smartphone addiction. The usage of mobile messengers by teenagers was followed by Internet browsing, gaming, and social networking service use for the longest periods of time [7]. With the Coronavirus Disease 2019 (COVID-19) outbreak, more people worldwide are playing video games and using the Internet. As a result, worries regarding teenagers acquiring behavioral addiction have been raised. The majority of teenagers have smartphones and access to the Internet [8]. Therefore, it is of interest to document the smartphone obsession linked behavioural changes among Indian adolescents.

## Methodology:

A School based, co relational study was conducted among 100 adolescents at selected schools at Tamil Nadu, India. Simple random selection techniques were used to choose samples from those who met the study's inclusion requirements. Adolescent aged 12-19 years who were studying 9th-12thstandard at selected schools from Tamil Nadu.

## The instrument used in the study had three parts:

Part 1: Socio Demographic Variables of the samples. It consists of 15 items related to their details Age, Gender, Standard, No of Siblings, Birth Order, Type of family, Religion, Area of Living, Duration of Mobile phone usage, Purpose of mobile phone usage

Part 2: Smartphone Addiction Scale-Short Version (SAC-SV) was used to assess the Smartphone Obsession. Ten items, ranging from 1 "strongly disagree" to 6 "strongly agree," are included. The overall score can vary from 10 to 60, with 60 representing the highest level of "Smartphone Obsession" over the previous year. The original SAS-SV demonstrated internal consistency, concurrent validity, and content validity.

Part 3: The self-report approach was utilized to assess the behavioral changes using the pediatric symptom checklist, version 17 (PSC-17). Each item is given a score of 0, 1, or 2 depending on whether it is "NEVER," "SOMETIMES," or "OFTEN" present. The sum of the scores for the 17 items is used to determine the final score. A high PSC-17 score of above 15. Both descriptive and inferential statistics were used in the data analysis.

## Results:

Table 1 shows that the participant's average age was 16.15 years, whereas the teenagers' average age ranged from 15 to 17 years. 58 (58%) of the 100 adolescents were male, and 42 (42%) were female. The majority of the 73 participants (73%) were in grades 9-10, and 52 (52%) of the teenagers had at least one sibling. Nearly 54 (54%) of the participants were their family's firstborn. 36 (36%) and 79 (79%) of the teenagers were raised in combined families, respectively. Eighty-one (81%) of the teenagers adhered to the Hindu religion, and 78 (78%) lived in rural areas.58 (58%) of the teenagers used their mobile phones continuously for 3-4 hours each day. The majority of teenagers 33 (33%) were using their smartphones for social media, while 31 (31%) were using them for communication.

**Figure 1:**
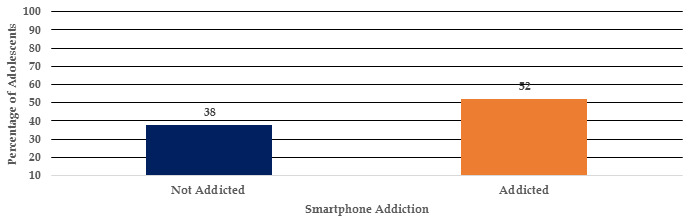
Level of smartphone obsession among adolescents (n=100)

Figure 1 shows the Smartphone Addiction Scale - Short Version (SAC - SV) was used to test adolescent smartphone Obsession; those who scored ≤ 30 were not addicted, and those who scored ≥ 30 were addicted. As a result, 62 (62%) students were to be addicted, compared to 38(38%) adolescents who were not.

Table 2 shows according to the item analysis results, the majority of participants have trouble focusing in class, (2.68 ± 1.45), and they won't be able to live without their smartphones (2.55 ± 1.65). However, the least number of participants (1.14 ± 0.89) said they would never stop using their smartphone, even if it had a significant negative impact on their daily lives.

Table 3 shows Teenagers who scored 15 or more on the Pediatric symptom check list, which was used to assess the extent of behavioural changes among them, may need to be referred to a qualified medical or mental health expert. This suggests that 37 teenagers require professional mental health counseling because 63 (63%) adolescents had desirable behavioural Change, whereas 37 37(37%) adolescents experienced undesirable behavioural change.

**Figure 2:**
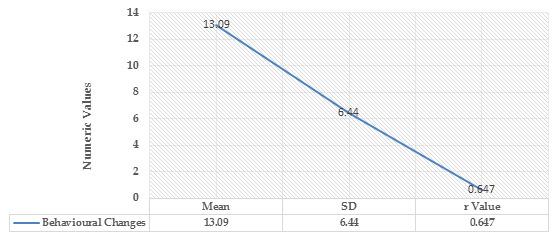
Correlation between smartphone Obsession with behavioural changes

Figure 2 shows a significant slight positive relationship was seen between smartphone addictions on behaviour changes, P < 0.001, which states that teenagers who used their smartphones more frequently exhibited undesirable behaviour.

## Discussion:

Another study from China demonstrates that Parental overprotection had an effect on the indirect pathway from parental smartphone addiction (PSA) to adolescent smartphone addiction (ASA) through the parent-child link (B = -0.016, p 0.001), whereas parental care had no effect (B = -0.005, p > 0.05). Parental overprotection in particular favorably regulated the second half mediation path [9]. A Cross sectional study shows Cell phone addiction was present in 33.0% (95% CI: 27.2-38.6) of the population. Girls (32.3%) and boys (33.6%) both had higher rates of addiction (p=0.835). Teenagers with three or fewer siblings, those who grew up in nuclear families, and those who began using mobile phones later than age 16 were all found to have much higher rates of cell phone addiction [1]. Christoph Randler *et al.* had focused on two distinct tests to determine smartphone addiction. In Study 1, 342 younger adolescents (13.39 1.77; 176 males, 165 girls, and 1 not specified) completed the Smartphone Addiction Proneness Scale (SAPS), while in Study 2, 208 older adolescents (17.07 4.28; 146 girls and 62 boys) completed the Smartphone Addiction Scale. Girls are more likely to develop smartphone addiction than boys, and gender is a significant predictor of addiction [10]. The experimental study evaluated the effects of complete smartphone abstinence versus a daily smartphone usage reduction by one hour on well-being and a healthy lifestyle. Participants in Germany used smartphones (N total = 619). Four measurement time points were used to assess variables like smartphone use (time, intensity, and problematic tendencies), life satisfaction, depressive symptoms, anxiety symptoms, physical activity, and smoking behavior. [11]. 31.33% of the sampled students have smartphone dependence. It was substantially correlated with gender (p=0.003, OR=1.91, CI: 1.23-2.99), family type (p=0.0012), kind of mobile phone used (p=0.001, OR=2.6, CI: 1.63-4.35), average daily mobile phone usage (p=0.001), and years of mobile phone usage (p=0.004, OR=2.4, CI: 1.31-4.55). In terms of public health, mobile phone dependence has been identified as a new issue [12]. The majority of respondents-84%-used smartphones, with the top three uses being phoning friends and family (96%), using the Internet, especially for social networking (91%), and using it for studying (78%)[13]. Another study from Uttarakhand found that 125 (43.90%) participants had lower academic performance due to excessive mobile phone use than 156 (54.70%) participants who had low mobile reliance, 191 (67%) participants who had negative behavioural changes, and 156 (54.70%) participants who had low mobile dependency. The overuse of mobile phones explained 49% of the variations among adolescents with certain factors, according to regression analysis [14].

## Conclusion:

Adolescents' dependence on mobile devices has emerged as a public health issue. Almost fifty percent of the teenagers in this study were also dependent on their phones, which has an impact on their behaviour. As a top priority, the Indian government should create measures to increase awareness of this issue for the benefit of young people's futures. In order to maintain adolescents' mental health, more needs to be done to design recreational programmes and include them actively.

## Figures and Tables

**Table 1:** Frequency and percentage distribution of socio demographic variables of adolescents (n=100)

**S.No**	**Variables**	**Frequency (%)**
1	Age in years	
	14-Dec	5 (5)
	15-17	87 (87)
	18-19	7(7)
2	Gender	
	Male	58 (58)
	Female	42 (42)
3	Standard	
	09-Oct	73 (73)
	11-Dec	27 (27)
4	No of Siblings	
	0	22 (22)
	1	52 (42)
	2	26 (26)
	>2	0
5	Birth Order	
	First Child	54 (54)
	Second Child	34 (34)
	Third Child	12(12)
6	Type of family	
	Joint	36 (36)
	Nuclear	59 (59)
	Extended	5 (5)
7	Religion	
	Hindu	81 (81)
	Muslim	9 (9)
	Christian	10(10)
	Others	0
8	Area of Living	
	Rural	78 (78)
	Urban	22 (22)
9	Duration of smart phone usage continuously	
	< 1 hr	2 (2)
	1- 2 hrs	35 (35)
	3 - 4 hrs	58 (58)
	> 4 hrs	5 (5)
10	Purpose of smart phone usage	
	Communication	31 (41)
	Social Media	33 (43)
	Education	14 (04)
	Entrainment	12 (12)
	Games	10 (10)

**Table 2:** Item wise analysis of smart phone Obsession among adolescents (n=100)

**S. No**	**Items**	**Mean ±SD**
1	Missing planned work due to smartphone use	1.46 ± 1.13
2	Having a hard time concentrating in class, while doing assignments, or while working due to smartphone use	2.68 ± 1.45
3	Feeling pain in the wrists or at the back of the neck while using a smartphone	2.25 ± 1.31
4	Won't be able to stand not having a smartphone	2.55 ± 1.65
5	Feeling impatient and fretful when I am not holding	1.79 ± 1.14
6	Having my smartphone in my mind even when I am not using it	1.67 ± 0.95
7	I will never give up using my smartphone even when my daily life is already greatly affected by it	1.14 ± 0.89
8	Constantly checking my smartphone so as not to miss conversations between other people on Twitter or Facebook	2.14 ± 1.62
9	Using my smartphone longer than I had intended	3.42 ± 1.85
10	The people around me tell me that I use my smartphone too much	1.51 ± 1.13

**Table 3:** Level of Behavioural Changes among adolescents (n=100)

**S. No**	**Level of Behavioural Changes**	**Score**	**Frequency (%)**
1	Undesirable	15-34	37 (37)
2	Desirable	0-14	63 (63)
